# Maraviroc (MVC) increases CD4+ and CD8+ cells: long-term data from the MVC clinical development program

**DOI:** 10.1186/1758-2652-13-S4-O45

**Published:** 2010-11-08

**Authors:** A Lazzarin, JG Sierra-Madero, M Battegay, G Mukwaya, R Burnside, D Chapman, J McCracken, S Portsmouth, S Valluri, H Valdez, J Heera

**Affiliations:** 1 San Raffaele Scientific Institute, Milano, Italy; 2 Dept. of Immunology & Infectious Diseases INNSZ, Mexico City, Mexico; 3 Univ. Hosp, Basel, Switzerland; 4 Pfizer Inc, New York, USA; 5 Pfizer Global Research, New London, USA; 6 Pfizer Inc, New York, USA; 7 Pfizer, 50 Pequot Ave, New London, USA

## Background

Across the MVC development program, patients who received MVC-containing regimens experienced greater increases in CD4+ cell counts than those observed in comparator arms in primary analyses. Here we present longer term immunological data from this program.

## Material and methods

Long-term (96 week) data from subjects with CCR5-tropic HIV infection in the following ongoing MVC studies were included in the analysis: (1) MOTIVATE study (MVC QD and BID vs placebo (PBO), each combined with optimized background therapy in treatment-experienced [TE] subjects); and (2) MERIT study (MVC BID vs EFV, each combined with ZDV/3TC in treatment-naïve [TN] subjects). Additionally, interim week 24 data from the ongoing 96-week 1078 study (MVC QD vs TDF/FTC, each combined with ATV/r in TN subjects) were summarized. Descriptive statistics are presented for data at baseline and at week 96 (MERIT/MOTIVATE) or week 24 (1078) pertaining to change in CD4+ and CD8+ cells.

## Results

Greater increases in CD4+ cells in MVC-containing groups persisted through 96 weeks in the MOTIVATE and MERIT studies, and through 24 weeks in the 1078 study (Table 1). Similarly, changes in CD8+ cells from baseline to weeks 96 or 24 favored MVC-containing regimens in all three studies. In a combined LOCF analysis of patients from all three studies at week 24, CD4+ counts increased by a median 100.5 cells/µL in 1260 recipients of MVC-containing regimens, compared with 84.5 cells/µL in 631 recipients of comparator regimens; CD8+ counts increased by a median 153 cells/µL in the MVC group, compared with a decrease of 61 cells/µL in the comparator group. At week 96, a combined analysis of patients from the MOTIVATE and MERIT studies showed median CD4+ increases from baseline of 129.5 and 100.3 cells/µL in the MVC (N=1177) and comparator (N=554) groups, respectively; CD8+ changes were 96 and -72 cells/µL, respectively. In all studies, differences in CD4+ and CD8+ counts between treatment groups were independent of differences in viral load changes (data not shown).

**Table 1:** 

Population	Treatment-experienced			Treatment-naive			
**Study**	**MOTIVATE – 96 Weeks**			**MERIT – 96 Weeks**		**1078 – 24 Weeks**	

**Arm**	**MVC QD**	**MVC BID**	**PBO**	**MVC BID**	**EFV**	**MVC QD**	**TDF/FTC**

**N**	**414**	**426**	**209**	**360**	**361**	**60**	**61**

BL HIV RNA (median log_10_ cp/mL)	4.86	4.85	4.86	4.88	4.85	4.59	4.66

Baseline CD4 count (median cells/µL)	171	167	171	244.3	258.5	344.5	358.0

Baseline CD8 count (median cells/µL)	867.5	836.5	820.8	791.5	860.0	859.8	890.0

CD4 change from BL (median cells/µL)	89.0	112.5	21.0	224.3	195.0	195.3	173.0

CD8 change from BL (medican cells/µL)	138.0	157.0	31.5	-3.00	-94.5	4.5	-78.5

## Conclusions

Greater increases in CD4+ and CD8+ counts were consistently achieved with MVC-containing regimens, compared to regimens without MVC, and persisted through at least 2 years of therapy in both TN and TE patients. These differences were independent of changes in HIV-1 RNA and are evident with multiple different MVC-containing regimens.

